# A bibliometric review of predictive modelling for cervical cancer risk

**DOI:** 10.3389/frma.2024.1493944

**Published:** 2024-11-19

**Authors:** Francis Ngema, Bonginkosi Mdhluli, Pako Mmileng, Precious Shungube, Mokgoropo Makgaba, Twinomurinzi Hossana

**Affiliations:** Centre of Applied Data Science, University of Johannesburg, Johannesburg, South Africa

**Keywords:** cervical cancer, risk prediction, machine learning, artificial intelligence, thematic analysis, natural language processing, latent Dirichlet allocation, predictive modelling

## Abstract

Cervical cancer represents a significant public health challenge, particularly affecting women's health globally. This study aims to advance the understanding of cervical cancer risk prediction research through a bibliometric analysis. The study identified 800 records from Scopus and Web of Science databases, which were reduced to 142 unique records after removing duplicates. Out of 100 abstracts assessed, 42 were excluded based on specific criteria, resulting in 58 studies included in the bibliometric review. Multiple scoping methods such as thematic analysis, citation analysis, bibliographic coupling, natural language processing, Latent Dirichlet Allocation and other visualisation techniques were used to analyse related publications between 2013 and 2024. The key findings revealed the importance of interdisciplinary collaboration in cervical cancer risk prediction, integrating expertise from mathematical disciplines, biomedical health, healthcare practitioners, public health, and policy. This approach significantly enhanced the accuracy and efficiency of cervical cancer detection and predictive modelling by adopting advanced machine learning algorithms, such as random forests and support vector machines. The main challenges were the lack of external validation on independent datasets and the need to address model interpretability to ensure healthcare providers understand and trust the predictive models. The study revealed the importance of interdisciplinary collaboration in cervical cancer risk prediction. It made recommendations for future research to focus on increasing the external validation of models, improving model interpretability, and promoting global research collaborations to enhance the comprehensiveness and applicability of cervical cancer risk prediction models.

## 1 Introduction

Cervical cancer represents a significant public health challenge, particularly affecting women's health globally (Ding et al., 2021). This challenge arises from various factors, including limited access to screening and early detection services, inadequate healthcare infrastructure, low levels of disease awareness, and persistent socioeconomic disparities. Moreover, the high prevalence of human papillomavirus (HPV) infection, the primary cause of cervical cancer, coupled with insufficient HPV vaccination coverage, contributes to elevated incidence rates (WHO, 2024). To effectively combat this challenge, a comprehensive approach is imperative, involving strengthening healthcare systems, expanding access to affordable screening and vaccination programs, and implementing extensive education and awareness initiatives.

The Sustainable Development Goals (SDGs) adopted by United Nations member states highlight the importance of reducing premature mortality from non-communicable diseases (StatsSA, 2023). Efforts to address this align with the World Health Organisation's (WHO) call to action in 2018 to eliminate cervical cancer as a public health concern (Gultekin et al., 2020). WHO's strategy aims to increase HPV vaccination uptake and screening coverage to achieve a significant reduction in cervical cancer incidence rates.

Cervical cancer screening plays a pivotal role in reducing morbidity and mortality associated with the disease. Traditional methods like the Pap smear have evolved into more advanced techniques such as liquid-based cytology (LBC), high-risk HPV (hrHPV) testing, and artificial intelligence (AI)-powered systems (Swanson and Pantanowitz, 2024). These advancements aim to overcome limitations of sensitivity and accuracy associated with traditional approaches, particularly in resource-limited settings.

While traditional cervical cancer prediction models rely on established risk factors and statistical methods, machine learning (ML) techniques offer innovative solutions by using complex algorithms and large datasets (Liu et al., 2023; Meng et al., 2022; Hu et al., 2018). ML algorithms, including support vector machines, random forests, and deep learning networks, have shown promise in enhancing sensitivity, specificity, and overall accuracy in predicting cervical cancer risk (Rahimi et al., 2023; Zhang et al., 2020; Esteva et al., 2019). Challenges such as model interpretability and data bias remain significant concerns in ML application to cervical cancer prediction (Singh and Goyal, 2020).

To gain a comprehensive understanding of the current state of research and identify potential future directions, a bibliometric analysis is a valuable tool. This approach allows for the systematic exploration of the intellectual landscape by analysing publication patterns, citations, keywords, and collaborations within a specific research field (Vargas-Cardona et al., 2023). Bibliometric analysis, a quantitative methodology, offers a powerful tool to explore the evolving knowledge structure within a specific research field (Jimma, 2023). This method provides researchers with a systematic approach to analysing publication patterns, citations, keywords, and collaborations.

Through investigating these aspects, bibliometric analysis yields measurable, accurate, and detailed information on the field (Donthu et al., 2021). This comprehensive understanding empowers researchers to not only gain a thorough grasp of the subject matter but also to foster a multidisciplinary approach, a key factor in advancing scientific progress (Motamedi et al., 2023).

In the context of cervical cancer risk prediction modelling, a bibliometric analysis can reveal how this field has developed over time, identify prominent researchers and institutions contributing to the field, and uncover emerging research trends. This knowledge will be crucial for guiding future research ventures and improving our ability to predict and prevent cervical cancer. In this review, the authors employed a bibliometric analysis along with other scoping methods to investigate the field of predictive modelling for cervical cancer risk assessment.

The aim of this study was to identify foundational literature and thematic content in cervical cancer risk prediction modelling through citation analysis, and to examine research trends, collaboration patterns, and niche areas to uncover gaps and propose new directions for future research.

The study contributes to cervical cancer research by enhancing understanding of cervical cancer risk prediction through comprehensive thematic analysis, revealing core themes, relationships, and broader trends. It advances methodological approaches by integrating Braun and Clarke's framework with Natural Language Processing (NLP). The study identifies emerging niche themes like “machine learning algorithms” and “predictive models”, and highlights a multidisciplinary approach involving machine learning, deep learning, and clinical validation.

These insights can facilitate interdisciplinary collaborations and improve prevention, diagnosis, and treatment outcomes in cervical cancer research. For example, the identification of emerging themes such as “machine learning algorithms” can lead to collaborations between data scientists, statisticians, computer scientists, pathologists, general practitioners and oncologists, resulting in the development of advanced predictive models that enhance early detection and personalised treatment plans for cervical cancer patients.

The remainder of the study is structured as follows: the next section details the methodology that guided the study. It is followed by the results and discussion section, the limitations, and the conclusion.

## 2 Materials and methods

This review uses bibliometric analysis, a method for studying scientific publications, to examine research on machine learning for cervical cancer risk prediction. It analyses the structure and content of existing research articles to understand how this field is developing. This review aims to provide a clear picture of current research trends and how machine learning is being used to improve cervical cancer risk assessment.

### 2.1 Data source and search strategy

The Population, Intervention, Comparison, and Outcome (PICO) framework was used to structure the search strategy. This is summarised in Table 1. This method ensured a comprehensive search strategy to gather relevant publications related to predictive modelling in cervical cancer risk assessment, covering key aspects such as population characteristics, intervention methods, comparison with traditional approaches, and outcome measures.

**Table 1:** Search terms.

**Concept**	**Search terms**
Population	“Cervical cancer” OR “Cervical cancer risk factors” OR “Uterine Cervical Neoplasms” OR “Cervical Intraepithelial Neoplasm” OR “CIN”
Intervention	“Machine learning algorithms” OR “Logistic regression” OR “Random forest” OR “Support Vector Machines (SVM)” OR “XGBoost” OR “Deep Learning” OR “Neural Networks” OR “Decision Tree” OR “Gradient Boosting” OR “KNN” OR “Naive Bayes”
Comparison	“Pap smear” OR “HPV testing” OR “VIA (Visual Inspection with Acetic Acid)” OR “histopathological examination (histology)” OR “Cytology” OR “Nomograms” OR “clinical prediction rules” OR “risk assessment scores” OR “Colposcopy”
Outcome	“Accuracy” OR “sensitivity” OR “specificity” OR “false positive rate” OR “AUC (Area Under Curve)” OR “predictive values” OR “calibration” OR “clinical utility” OR “clinical support decision”
Refined search strategy for Web of Science	(“machine learning” OR “predictive modelling” OR “artificial Learning”) NEAR/10 (“cervical cancer” OR “cervical risk”)
Refined search strategy for Scopus	(“machine learning” OR “predictive modelling” OR “artificial Learning”) W/10 (“cervical cancer” OR “cervical risk”)

### 2.2 Data collection, cleaning and organisation

#### 2.2.1 Data collection

**Figure 1:**
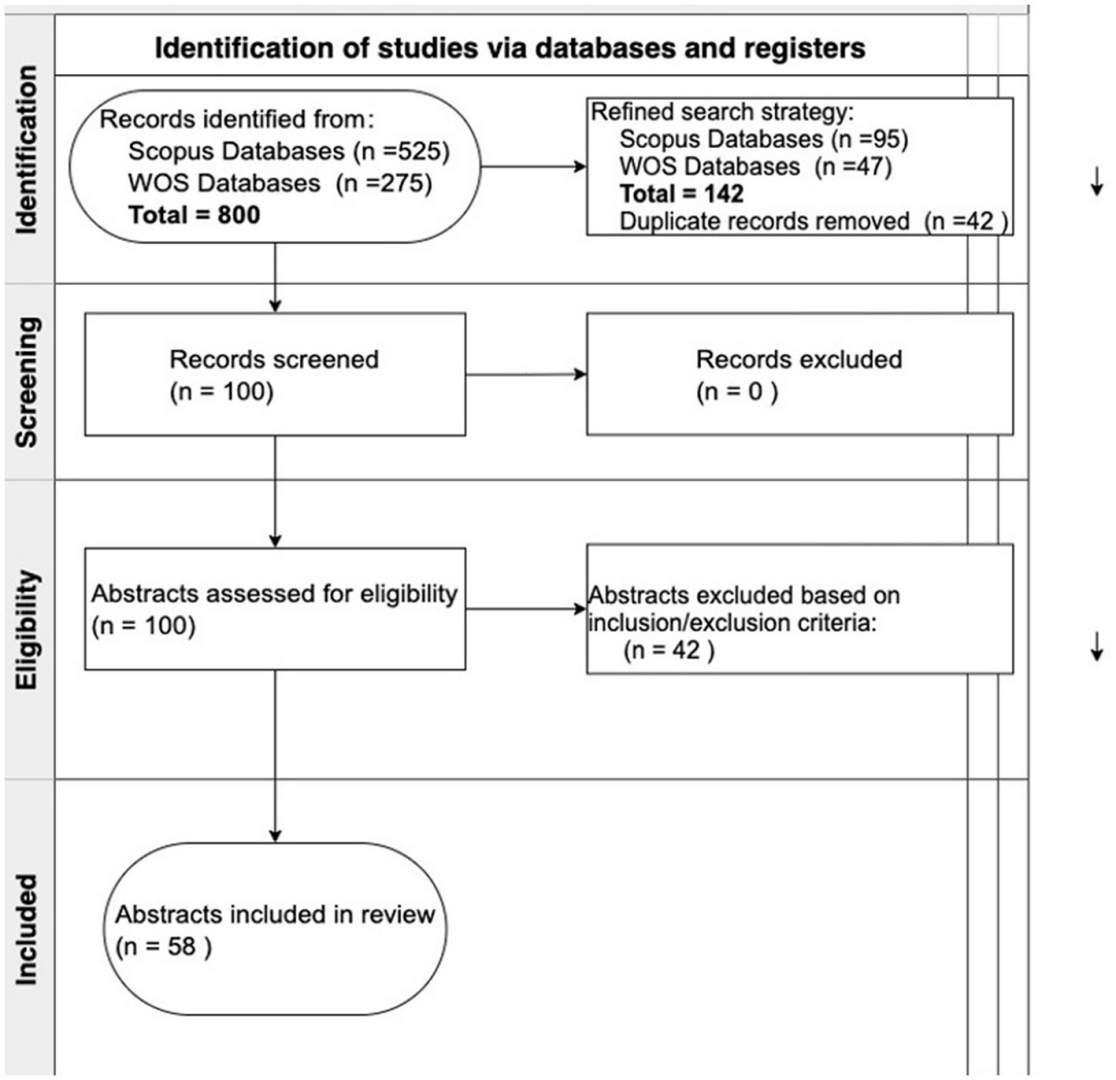
Flow diagram for the bibliometric review.

The entire data collection process is illustrated in a flowchart (see Figure 1). This flowchart visually depicts the number of records identified, screened, excluded, and ultimately included in the final analysis, promoting transparency and replicability of our research methods.

Both Scopus and Web of Science databases were used to retrieve relevant publications. Scopus was chosen for its comprehensive coverage across various disciplines, while Web of Science provided access to high-quality scholarly literature, ensuring a thorough exploration of the research landscape. The search strategy was refined by including proximity operators.

Proximity operators function as specialised search query keywords designed to refine retrieval by dictating the closeness of terms within a document (Goldman et al., 1998). Unlike Boolean operators that focus on presence or absence, proximity operators emphasise the conceptual proximity of search terms. This ensures retrieved documents not only contain the specified keywords but also discuss them in close association, enhancing the relevance of results.

In Web of Science, the NEAR/x operator dictates the maximum number of intervening words between terms (e.g., “cancer NEAR/3 risk”). Conversely, Scopus utilises W/x expressions, requiring terms to appear in the specified order within a defined word range (e.g., “Cervical Cancer” W/2 “Machine learning”). Researchers can significantly improve the focus and relevance of their literature searches within Web of Science and Scopus by strategically employing proximity operators within each platform's designated syntax.

The retrieved data was saved in various file formats, e.g., .csv and .bib. The software EndNote was employed to facilitate the management of articles from both databases. EndNote allows for efficient organisation and storage of citation information, aiding in the subsequent stages of the research process.

#### 2.2.2 Data cleaning and organisation

The retrieved publications from both databases underwent initial screening to identify and eliminate duplicates, thereby ensuring the integrity of the dataset. Subsequently, citation information and metadata were standardised and formatted uniformly to facilitate seamless analysis and comparison across datasets. The datasets obtained from Scopus and Web of Science were merged, and duplicates were subsequently removed using R Studio. As a result, the final dataset comprised 100 unique articles for further analysis.

The articles were distributed among five authors, with each responsible for reviewing twenty articles. Each publication underwent a thorough assessment for relevance and quality, guided by predefined inclusion and exclusion criteria outlined in Table 2. The authors convened to collectively discuss each article and reached a consensus on whether to include or exclude it based on the established criteria. Publications pertaining to treatment or involving patients already diagnosed with cervical cancer were excluded from the final dataset. Consequently, the refined dataset consisted of 58 distinct articles selected for further analysis.

**Table 2:** Inclusion/exclusion criteria.

Relevance	The article specifically addressed the development, validation, or application of predictive models for assessing cervical cancer risk. Studies investigating other aspects of cervical cancer, such as treatment or diagnosis, were excluded. Duplicate publications (preference given to the most comprehensive or recent publication) were excluded.
Study design	Peer-reviewed original research articles, systematic reviews, meta-analyses, and clinical trials were included. These studies provided robust evidence for the effectiveness of predictive models. Non-research articles including editorials, commentaries, letters, conference abstracts, and case reports were excluded. Studies with limited methodology or unclear results (e.g., lacking clear descriptions of the model, data analysis, or with inconclusive findings) were excluded.
Population	Studies involving human subjects of any age, ethnicity, or geographical location were included. This ensured a diverse representation of the population at risk for cervical cancer. Studies focusing solely on animals or *in vitro* models were excluded, as these findings did not translate directly to human risk assessment.
Predictive models	Articles that described the development, validation, or application of predictive models for assessing cervical cancer risk. The type of model (e.g., machine learning algorithm, statistical model) was not a limitation. Articles describing predictive models for diseases other than cervical cancer were excluded to maintain focus.
Outcome measures	Studies reporting on the performance metrics of the predictive models for cervical cancer risk assessment were essential. This included measures like sensitivity, specificity, and area under the receiver operating characteristic curve (AUC). Studies lacking performance metrics or validation of the predictive models were excluded.
Language	Articles published in English were preferred due to wider accessibility. However, articles in other languages were considered where translated versions were available.
Publication date	No restriction was applied to the publication date. This allowed for the inclusion of both recent advancements and established research in the field.

The accuracy of citation details and publication metadata was verified to minimise errors and inconsistencies in the dataset.

### 2.3 Bibliometric analysis

Science mapping, as defined by Donthu et al. (2021) examines the relationships between research constituents, focusing on intellectual interactions and structural connections among them. This analysis employed techniques such as citation analysis, thematic analysis, bibliographic coupling, co-word analysis, and co-authorship analysis (Donthu et al., 2021; Öztürk et al., 2024). When combined with network analysis, these techniques were instrumental in presenting the bibliometric structure and the intellectual structure of the research field.

#### 2.3.1 Citation analysis

Citation analysis was utilised to identify highly cited publications and uncover their thematic content (Khare and Jain, 2022). This involved examining the number of citations received by each publication to gauge its impact in the field. Relevant bibliometric software, such as VOSviewer and BiblioShiny, were employed to analyse citation data and visualise citation networks, aiding in the identification of influential publications and their intellectual connections.

#### 2.3.2 Thematic analysis

This research approach merged the strengths of thematic analysis with the power of Natural Language Processing (NLP) techniques. Braun and Clarke's thematic analysis framework provided a systematic approach for identifying, analysing, and reporting patterns within qualitative data (Öztürk et al., 2024). Thematic analysis, a qualitative analysis method, involves coding documents to identify themes and capturing qualitative attributes relevant to the research subject (Braun and Clarke, 2006).

Initially, the dataset underwent Term Frequency-Inverse Document Frequency (TF-IDF) vectorisation and stop word removal to preprocess the text, followed by the application of Latent Dirichlet Allocation for topic modelling to identify five topics within the abstracts. The second approach employed Generate Similar (Gensim) dictionary to convert the text data into a bag-of-words representation, followed by LDA-based topic modelling configured to identify 6 topics. LDA is a statistical technique that identifies latent thematic structures within a collection of documents (Jelodar et al., 2019).

Two approaches were combined for examining the abstract text data utilising LDA to identify topics within the abstracts, with the LDA model extracting the most prominent words associated with each topic, revealing prevalent themes related to cervical cancer risk prediction modelling. Subsequently, the topics from both approaches were merged to form four overarching themes.

The thematic analysis process involved familiarisation with the data, followed by documenting and coding, similar to tokenisation. Irrelevant words were removed to focus on pertinent information. Themes were generated by observing patterns in the codes and assessing their correlation, ensuring alignment with the research objectives. The iterative review of themes led to their refinement and finalisation, ultimately contributing to the clarification of prevalent themes within the abstracts pertaining to cervical cancer risk prediction modelling.

However, to gain a more comprehensive understanding, three complementary methods were used: thematic mapping, thematic evolution analysis, and trend topic analysis. Thematic mapping involves visually representing the relationships between identified themes, creating a map-like depiction that clarifies how themes interact and influence each other (Agbo et al., 2021). This visual representation enhances our comprehension of the thematic landscape and the interplay of themes within the data. Using BiblioShiny, a software program for analysing research publications, this study was able to identify how the main themes in cervical cancer research have shifted focus over time.

This revealed interesting trends, such as one topic becoming less popular while another gained more attention (Khare and Jain, 2022). This analysis also revealed the emergence of entirely new themes within the data. Finally, trend topic analysis complemented thematic analysis by exploring broader trends within the data. This involved analysing the frequency of specific terms or concepts to identify areas of growing interest or decline over time (Liang et al., 2021).

#### 2.3.3 Bibliographic coupling

Bibliographic coupling was employed to identify recent and niche publications that share common references. This technique is based on the assumption that scholarly articles that cite a significant number of the same sources are likely to be similar in their content and research focus (Donthu et al., 2021). The study analysed a dataset of 48 articles and created a visual representation to explore these connections.

Due to VOSviewer's criteria for bibliographic coupling, only 48 out of the 58 articles were included in the visualisation of research trends. This visualisation helped to understand the thematic landscape and how prominent themes connect with specific research fields and journals. Researchers can identify thematic clusters within a body of literature by analysing these citation patterns.

#### 2.3.4 Data analysis and visualisation

BiblioShiny was used to analyse publication trends and geographic distribution of research output relevant to cervical cancer risk prediction. VOSviewer complemented this by creating visualisations like citation networks and thematic maps. These techniques helped interpret results, identifying key themes, research gaps, emerging trends, and prominent research institutions/authors in cervical cancer risk prediction research.

## 3 Results and discussions

Table 3 illustrates the data, comprising 58 articles sourced from 49 publications, exhibited a robust annual growth rate of 13.43%, reflecting a growth trend. The average document age of 2.97 years and an average citation count of 14.84 per document further highlighted the recent nature and influential reach of these publications.

**Table 3:** Overview of the dataset.

**Description**	**Results**
**Main information about data**	
Timespan	2013:2024
Sources (journals, books, etc.)	49
Documents	58
Annual growth rate %	13.43
Document average age	2.97
Average citations per doc	14.84
References	86
**Document contents**	
Keywords plus (ID)	665
Author's keywords (DE)	180
**Authors**	
Authors	288
Authors of single-authored docs	1
**Authors collaboration**	
Single-authored docs	1
Co-authors per doc	5.28
International co-authorships %	0
**Document types**	
Article	58

Collaboration is a defining characteristic of authorship on the prediction of cervical cancer, with an average of 5.28 co-authors per document. The study observed a significant trend of interdisciplinary collaboration in the field of cervical cancer risk predictive modelling. This collaboration was characterised by the integration of expertise from diverse academic domains, including computer science, mathematics, statistics, biomedical engineering, epidemiology, oncology, pathology, obstetrics, gynaecology, and public health. International co-authorships were absent, suggesting potential avenues for expanding research collaborations and fostering global knowledge exchange in cervical cancer research.

Notably, only one document had a single author, further emphasising the collaborative nature of research in this domain. As all documents are classified as articles, the data underscores the emphasis on scholarly rigour and methodological depth within cervical cancer research. These findings highlighting the importance of interdisciplinary collaborations and innovative discoveries in the fight against predictive cervical cancer. For example, one highly cited work documented a successful collaboration between computer scientists, biomedical engineers, pathologists, and oncologists. This collaboration resulted in a computer-aided diagnosis (CAD) system for cervical cancer screening. The system achieved high accuracy in detecting and classifying cervical tissue abnormalities, potentially leading to improved patient outcomes and increased survival rates for women (Alquran et al., 2022).

### 3.1 Citation analysis

The top 20 highly cited research articles presented in Table 4 explored various methods for improving cervical cancer screening, diagnosis, and risk prediction. Machine learning algorithms were a common theme, with studies applying them to analyse clinical data, Pap smear images, and self-collected samples. These approaches have achieved high accuracy in some cases, exceeding the performance of traditional methods.

**Table 4:** Top 20 highly cited publications.

**Rank**	**Title**	**Year**	**Journal**	**Study objectives**	**Methodologies**	**Key findings**	**Citation count**	**Focus area**
1	Datadriven cervical cancer prediction model with outlier detection and oversampling methods	2020	Sensors	Propose a cervical cancer prediction model using risk factors as inputs.	Uses DBSCAN & IForest for outlier detection, SMOTE & SMOTE Tomek for balancing, Random Forest for classification.	IForest with SMOTE and IForest with SMOTETomek outperform other methods; RF is best classifier. CCPM shows better accuracy than previous methods.	145	Cervical cancer prediction
2	Hybrid model for detection of cervical cancer using causal analysis and machine learning techniques	2022	Comp Math Methods Med	Efficient feature selection and prediction model for cervical cancer datasets.	Boruta analysis for feature selection and SVM for classification.	Boruta with SVM outperforms existing methods.	70	Cervical cancer classification
3	A comprehensive study on multiclass cervical cancer diagnostic prediction on Pap smear images	2020	Tissue Cell	Multi-class diagnosis of cervical lesions using deep learning.	Evaluates AlexNet, VGGNet (VGG-16 & VGG-19), ResNet (ResNet-50 & ResNet-101) and GoogleNet architectures.	Ensemble classifier with three best deep learning models achieves high accuracy multi-class classification.	60	Cervical cancer diagnosis
4	Cervical cancer diagnosis based on random forest	2017	Int J Perform Eng	Framework for cervical cancer diagnosis using Random Forest and feature selection.	Uses pre-processing, segmentation, relief for feature selection, and RF for classification.	RF with top 13 features outperforms other classifiers with 94.44% accuracy.	56	Cervical cancer diagnosis
5	Exemplar pyramid deep feature extraction based cervical cancer image classification model using papsmear images	2022	Biomed Signal Process Control	Detects cervical cancer using exemplar pyramid deep feature extraction method.	Employs transfer learning with Darknet19 or Darknet53 in an exemplar pyramid structure and Neighborhood.	Achieves 98.26 and 99.47% accuracy on Sipakmed and Mendeley LBC datasets, respectively.	53	Cervical cancer detection
6	Machine learning-based statistical analysis for early stage detection of cervical cancer	2021	Comput Biol Med	Find efficient models to detect early-stage cervical cancer using clinical data.	Apply random forest (RF), and instance-based K-nearest neighbor (IBK) to four types of clinical data.	RF performs best for biopsy and cytology, RF and IBK perform best for Hinselmann (99.16%) and Schiller (98.58%) data, respectively.	39	Cervical cancer detection
7	Cervical cancer diagnostics using hybrid object detection adversarial networks	2022	IEEE J Biomedical Health Inform	FSOD-GAN for screening and diagnosing cervical cancer using colposcopy images.	Faster R-CNN for spot detection and hierarchical classification of cancer stages.	Achieves 99% accuracy in diagnosing cervical cancer stages.	34	Cervical cancer screening and diagnosis
8	DCAVN cervical cancer prediction using deep convolutional and variational autoencoder	2021	Multimedia Tools Appl	Automate cancer diagnosis and classification with deep learning techniques.	Uses CNN with variational autoencoder for data classification.	Outperforms traditional methods with 99.2 and 99.4% accuracy.	30	Cervical cancer diagnosis
9	Performance analysis of machine learning algorithms for cervical cancer detection	2020	Int J Healthcare Information Systems and Informatics	Apply ML algorithms for cervical cancer detection.	Uses segmentation, Extra Tree for feature selection, and logistic regression with L1 regularization.	Achieves up to 100% accuracy on some datasets.	27	Cervical cancer detection
10	Cervical cancer classification using combined ML and deep learning approach	2022	Comput Mater Continua	Develop a computer-aided diagnosis system to classify Pap-smear images.	ResNet101 for feature extraction and SVM for classification.	Achieved 100% accuracy for distinguishing normal/abnormal cases.	27	Cervical cancer screening (deep learning)
11	Genome-wide miRNA analysis of HPV-positive self-samples for early detection of cervical cancer	2019	Int J Cancer	Identify deregulated miRNAs as triage markers for cervical cancer in self-samples.	Uses small RNA sequencing and qPCR for validation.	Identifies a 9-miRNA marker panel with AUC of 0.89 for CIN3 detection.	26	Cervical cancer screening (self-sampling)
12	Predicting cervical cancer using machine learning methods	2020	Int J Adv Comput Sci Appl	Develop ML model for accurate cervical cancer diagnosis.	Uses voting classifier, SMOTE, and PCA on cervical cancer risk factor dataset.	Achieves higher accuracy and sensitivity than cytology alone.	25	Cervical cancer diagnosis
13	Real world effectiveness of primary screening with high risk human papillomavirus testing in the cervical cancer screening program in china a nationwide population based study	2021	BMC Med	Evaluate HPV testing effectiveness in cervical cancer screening.	Population-based study comparing HPV testing and cytology.	HPV testing has higher detection rate and predictive value for CIN2+.	24	Cervical cancer screening (HPV testing)
14	Development of a cervical cancer progress prediction tool for human papillomavirus positive Koreans	2015	J Int Med Res	Develop web-based tool to predict high-risk cervical lesions in HPV positive women.	Use Support Vector Machine (SVM) model to identify patient features using PAP smear and HPV genotype data.	Achieves 74.41% accuracy using four selected features (PAP, HPV16, HPV52, and HPV35).	18	Cervical cancer risk prediction
15	A cervical abnormality risk prediction model can we use clinical information to predict which patients with ascuslsil pap tests will develop cin 23 or ais	2013	J Lower Genital Tract Dis	Model for predicting precancerous lesions in women with mild PAP abnormalities.	Multivariate logistic regression on clinical and demographic data.	Poor individual predictive ability due to data limitations.	17	Cervical cancer risk prediction
16	Ordinal losses for classification of cervical cancer risk	2021	Peerj Comput Sci	Improved cervical cancer risk prediction using ordinal loss in neural networks.	Non-parametric ordinal loss function for deep neural networks.	Achieves 75.6% accuracy for seven classes, 81.3% for four classes.	16	Cervical cancer screening (deep learning)
17	Hematological markers as predictors for cervical cancer	2019	J Oncol	Investigate hematological markers for cervical cancer diagnosis.	Analyzes Neutrophil-to-Lymphocyte Ratio (NLR) and Platelet-to-Lymphocyte Ratio (PLR).	NLR and PLR significantly elevated in cervical cancer patients.	15	Cervical cancer diagnosis (inflammatory markers)
18	A machine learning-based framework for the prediction of cervical cancer risk in women	2022	Sustainability	Develop a deep learning model for cervical cancer risk prediction using HPV test data.	Proposes a deep learning model; not fully implemented.	Promising future development for risk prediction.	11	Cervical cancer risk prediction (deep learning)
19	Hybridization of deep learning pretrained models with machine learning classifiers and fuzzy minmax neural network for cervical cancer diagnosis	2023	Diagn	Improve Pap-smear image classification accuracy using deep learning and fuzzy neural networks.	Combine deep learning architectures with fuzzy min-max neural networks to classify Pap-smear images.	Achieved 95.33% classification accuracy on Pap-smear image dataset.	11	Cervical cancer diagnosis (deep learning)
20	The role of high-risk human papilloma virus testing in the surveillance of cervical cancer after treatment	2023	Arch Pathol Lab Med	Investigate the role of HR-HPV testing in predicting cervical cancer recurrence.	Retrospective study on patients who underwent HR-HPV testing during cervical cancer surveillance.	Persistent HR-HPV infection is a risk factor for cervical cancer recurrence.	11	Cervical cancer progression prediction

Recent advancements in cervical cancer research are revolutionising screening, diagnosis, and risk prediction methods. Machine learning algorithms are at the forefront, with studies such as Ijaz et al. (2020) utilising them to analyse clinical data for early cancer detection. Deep learning, a specialised machine learning technique, is also proving valuable. Hussain et al. (2020) demonstrate its effectiveness in Pap smear image analysis and classification using convolutional neural networks.

**Figure 2:**
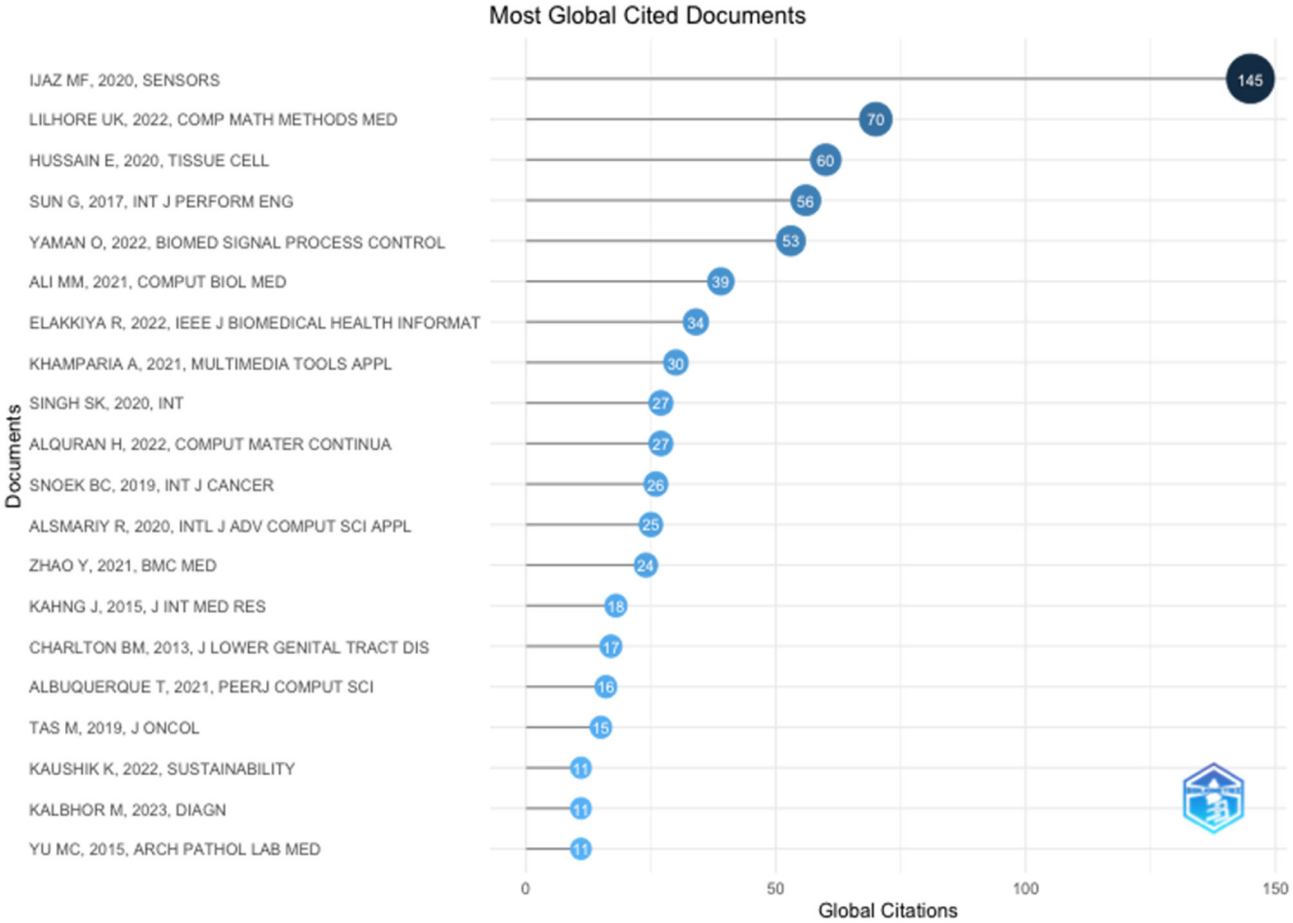
Top 20 most global cited articles.

Beyond machine learning, the table highlights other promising techniques. Yaman and Tuncer (2022) introduced a novel deep feature extraction method for accurate cancer detection, while Ali et al. (2021) showcased the efficacy of machine learning models in analysing various clinical datasets to identify early-stage cervical cancer. Additionally, research on HPV testing, a major risk factor, is ongoing, with studies exploring its potential to improve screening programs (Zhao et al., 2021). Furthermore, Figure 2 shows the most globally cited authors. Overall, these advancements offer significant hope for improved cervical cancer outcomes and enhanced patient care.

The top 20 highly cited publications in cervical cancer research revealed a multi-pronged approach using recent advancements. Machine learning and deep learning techniques were prominent, demonstrating effectiveness in analysing various data sources—clinical data, Pap smear images, and HPV test results. Algorithms such as Random Forest and convolutional neural networks achieved high accuracy in classification and detection tasks.

Furthermore, researchers developed methods for efficient feature selection and model optimisation (e.g., Boruta analysis) to refine these tools and enhance model performance. Early detection remains a critical focus, with novel models such as the Cervical Cancer Prediction Model utilising risk factors and clinical data for early identification. This focus aimed to enhance patient outcomes through timely intervention.

Enhanced screening strategies are another key theme. Integration of advanced technologies such as digital colposcopy and image analysis were explored to improve accuracy and efficiency. In addition, the role of HPV testing in identifying high-risk individuals was investigated for improved screening protocols. Finally, the importance of clinical validation and translation of research findings into practical applications was emphasised. Extensive validation studies ensure the generalisability of proposed methodologies, thus, bridging the gap between research and improved patient care.

Thematic content analysis revealed a multidisciplinary approach in cervical cancer research. Machine learning, deep learning, clinical validation, and translation contributed significantly to innovation in cervical cancer detection, diagnosis, and management.

The reviewed studies on machine learning in cervical cancer research reveal several gaps. Many studies lack external validation on independent datasets, limiting the robustness and generalisability of their findings. Most studies do not address model interpretability, crucial for understanding prediction mechanisms. Socioeconomic factors, such as income and access to healthcare, are largely ignored despite their significant impact on cervical cancer risk and outcomes. Furthermore, information on dataset size and diversity is often missing, hindering the assessment of model performance and applicability across different populations. Addressing these gaps is essential for advancing the practical application of machine learning in cervical cancer care.

### 3.2 Co-citation analysis

**Figure 3:**
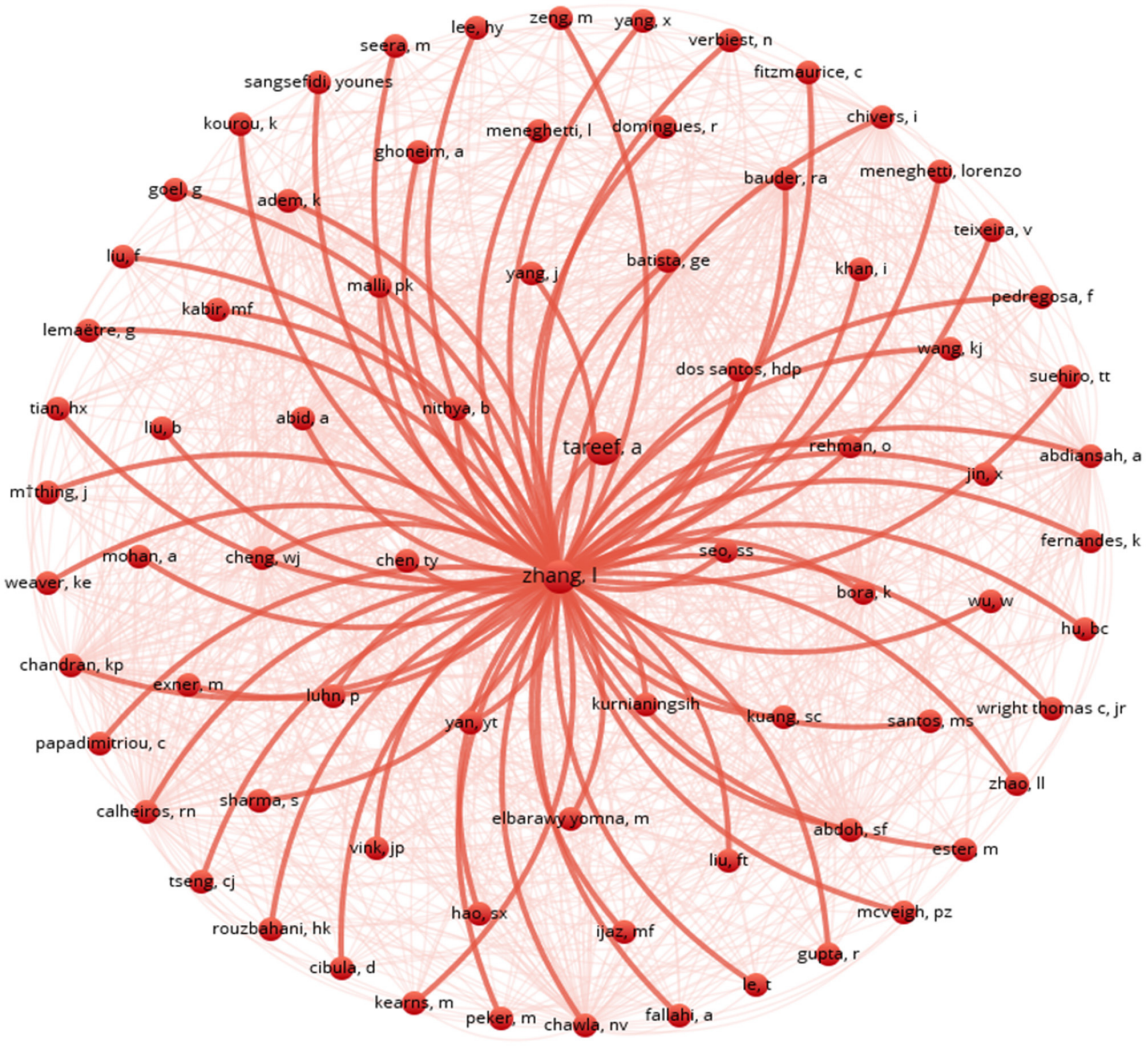
Co-citation network.

The visual representation in Figure 3 depicts a co-citation network with authors as nodes connected by lines indicating their co-citations, with the central node labelled “Zhang” prominently displayed. Surrounding this central node are numerous red lines connecting to other author nodes, suggesting that Zhang is highly cited with these authors. Other author nodes, labelled with various names or abbreviations, are scattered around the central node, with connections represented by red lines of varying opacities.

The denser lines indicate stronger co-citation relationships, resembling a starburst pattern emanating from the central “Zhang” node. This network structure implies that “Zhang” holds significant influence in the field, with the connections revealing collaborative relationships and influential connections among authors. Co-citation networks offer valuable insights into scholarly collaboration, research trends, and influential figures, aiding researchers in understanding the dynamics of scientific collaboration and impact.

### 3.3 Thematic analysis

The analysis of abstracts identified emerging themes within cervical cancer risk prediction modelling, represented by codes extracted from the text data. Table 5 summarises the frequency counts of these codes, showcasing the prominence of keywords such as “cervical,” “cancer,” “model,” “patient,” and “accuracy.” Additionally, codes such as “pap,” “study,” and “machine” were also prevalent, indicating themes related to diagnostic methods, research studies, and machine learning techniques, respectively. These codes provided valuable insights into the key concepts and areas of focus within the abstracts, facilitating a deeper understanding of the research landscape in cervical cancer risk prediction.

**Table 5:** Codes for emerging themes from the abstracts.

Word	cervical	cancer	model	patient	Accuracy	learning	feature	Image	result	method
Count	266	257	123	92	90	84	81	79	78	76
Word	pap	study	machine	smear	classification	woman	algorithm	Risk	hpv	diagnosis
Count	70	62	60	58	56	56	55	55	54	49

TF-IDF vectorisation and LDA produced topics characterised by prominent keywords, delineating prevalent themes within the abstracts. These topics encompassed a spectrum of concepts, with Topic 0 focusing on cancer and cervical, Topic 1 emphasising patient-related terms, Topic 2 highlighting medical images, Topic 3 focusing on patient-centred models, and Topic 4 showcasing features pertinent to predictive modelling. The weightings in Tables 6, 7 represent the probability of a word belonging to a specific topic, with higher weights indicating a stronger association.

**Table 6:** TF-IDF LDA results.

**Topic**	**Word 1**	**Word 2**	**Word 3**	**Word 4**	**Word 5**	**Word 6**	**Word 7**	**Word 8**	**Word 9**	**Word 10**
Topic 0	Cancer (0.029)	Cervical (0.028)	Model (0.013)	Learning (0.011)	Accuracy (0.011)	–	–	–	–	–
Topic 1	Feature	Model	Image	Propose	Accuracy	Pap	Patient	Classification	Smear	Dataset
Topic 2	HPV	Testing	Self	HR	Sample	Positive	Patient	Risk	Recurrence	Vs
Topic 3	Machine	Algorithm	Model	ML	Colposcopy	Algorithms	Result	Classifier	Colposcopic	Neural
Topic 4	CI	RT	Patient	AI	Nomogram	Lesion	Gynecologic	Grade	Associate	Prognosis
Topic 5	Recurrence	Adenocarcinoma	Invasion	Usual	Risk	Model	Tumour	Smote	Analyze	Month

**Table 7:** Gensim LDA results.

**Topic**	**Word 1**	**Word 2**	**Word 3**	**Word 4**	**Word 5**
Topic 0	Cancer (0.029)	Cervical (0.028)	Model (0.013)	Learning (0.011)	Accuracy (0.011)
Topic 1	Cervical (0.029)	Cancer (0.018)	Model (0.010)	Patient (0.010)	Accuracy (0.010)
Topic 2	Cancer (0.026)	Cervical (0.025)	Model (0.013)	Patient (0.008)	Image (0.008)
Topic 3	Cancer (0.022)	Cervical (0.020)	Model (0.010)	Patient (0.010)	Result (0.008)
Topic 4	Cancer (0.026)	Cervical (0.025)	Model (0.013)	Feature (0.013)	Patient (0.011)

Additionally, Gensim and LDA topic modelling revealed five key themes within cervical cancer risk prediction research. Topics 0 and 1 featured core concepts such as “cancer,” “cervical,” “model,” and “accuracy,” emphasising model development and machine learning. Topic 2 suggested the use of medical images for detection, while Topic 3 focused on patient-centred models with interpretable results. Topic 4 pointed to research on feature engineering for improved model performance, highlighting a strong focus on model development and translating research into practical clinical applications for improved patient outcomes.

This convergence of findings from both methodologies emphasises the multifaceted nature of cervical cancer risk prediction research. The clusters derived from both approaches included:

1. **Cervical cancer diagnosis and risk assessment with machine learning:** this cluster merged topics emphasising machine learning models for diagnosis and risk assessment in cervical cancer, incorporating both image-based and non-image-based approaches.
2. **HPV testing and risk factors for cervical cancer**: this cluster focused on the role of HPV testing, self-sampling methods, and their association with cervical cancer risk, potentially including comparisons with other risk factors.
3. **Prognosis and risk stratification in cervical cancer**: this cluster highlighted the importance of clinicopathological features, AI, and nomograms in determining a patient's risk grade and prognosis in cervical cancer.
4. **Cervical cancer recurrence analysis:** this cluster explored factors associated with cervical cancer recurrence, including tumour characteristics, risk models, and survival analysis methods, addressing data imbalance with SMOTE.

The integration of findings from both the Braun and Clarke thematic analysis and the NLP approach underscored the multifaceted nature of cervical cancer risk prediction research. The thematic and NLP analyses provided specific, actionable insights into cervical cancer risk prediction research. Thematic analysis revealed emerging themes such as diagnostic methods, machine learning techniques, and patient-focused models, underscored by the frequent occurrence of keywords like “cervical,” “cancer,” “model,” and “accuracy.”

NLP techniques like TF-IDF vectorisation and LDA highlighted key topics, including the development of predictive models, the use of medical images, and patient-centred approaches, as seen in the prevalence of terms across topics. This integrated methodology found research clusters such as machine learning for diagnosis, HPV testing, prognosis and risk stratification, and recurrence analysis. These findings emphasise the multidisciplinary nature of cervical cancer risk prediction, showing the combination of qualitative and quantitative insights to enhance understanding and guide future research directions.

**Figure 4:**
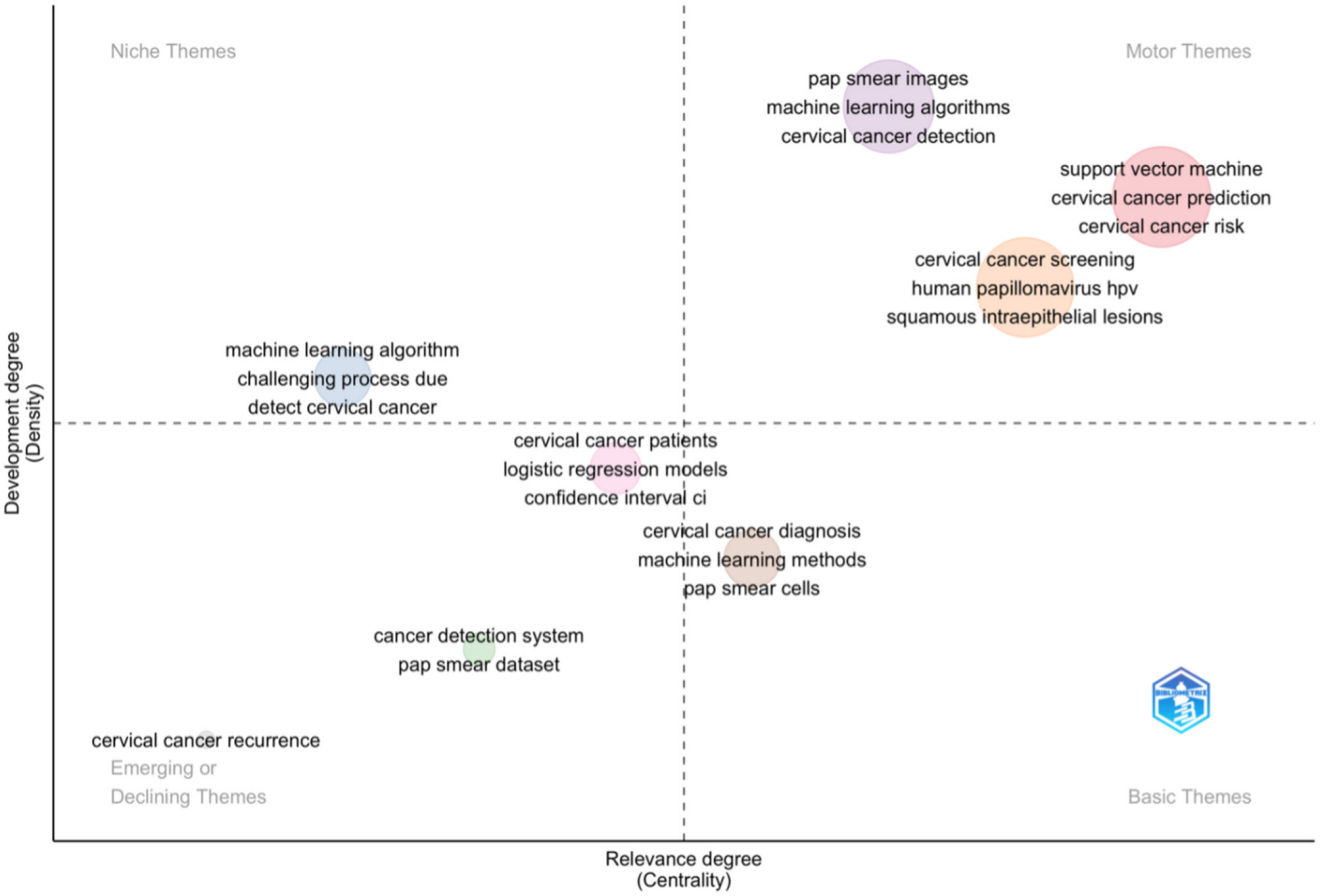
Thematic map.

The provided Figure 4 illustrates the thematic understanding of cervical cancer research and machine learning, aligning with the identified themes using BiblioShiny. A quadrant plot visualises the thematic landscape of cervical cancer research and machine learning. Two axes, developmental degree (vertical) and relevance degree (horizontal), divide the plot into quadrants. Niche themes, like “pap smear images” and “machine learning algorithms,” occupy a specific space, reflecting their focused nature. Conversely, central themes like “cervical cancer prediction” reside in the core area due to their high relevance.

Emerging or declining themes, such as “challenging process due to detecting cervical cancer,” are positioned accordingly. Established themes like “confidence interval (CI)” form the foundational “basic themes” quadrant. Specific terms like “support vector machine,” “human papillomavirus (HPV),” or “squamous intraepithelial lesions” further illustrate the thematic breakdown within each quadrant. The study gained insights into the relative importance and developmental stages of various research areas, ultimately aiding in identifying promising avenues and knowledge gaps within the field of cervical cancer research and machine learning by analysing this thematic distribution.

The study aligns with current trends, as commonly used models in cervical cancer risk prediction include support vector machine (SVM), random forest (RF), and multivariable logistic regression. This is further supported by research comparing machine learning algorithms for disease prediction, which identified SVM and RF as preferred algorithms (Uddin et al., 2019; Zhang et al., 2023; Yang et al., 2019).

The niche themes “detect cervical cancer” and “machine learning algorithms” signify specialised areas of focus within the broader research field. This means that there is a concentrated effort on using advanced computational techniques to improve the accuracy and efficiency of cervical cancer detection.

Researchers are increasingly integrating machine learning algorithms to develop innovative diagnostic tools, which can potentially enhance early detection, optimise treatment plans, and improve patient outcomes. This targeted research highlights the importance of interdisciplinary collaboration in advancing medical technologies and addressing specific challenges within cervical cancer care.

**Figure 5:**
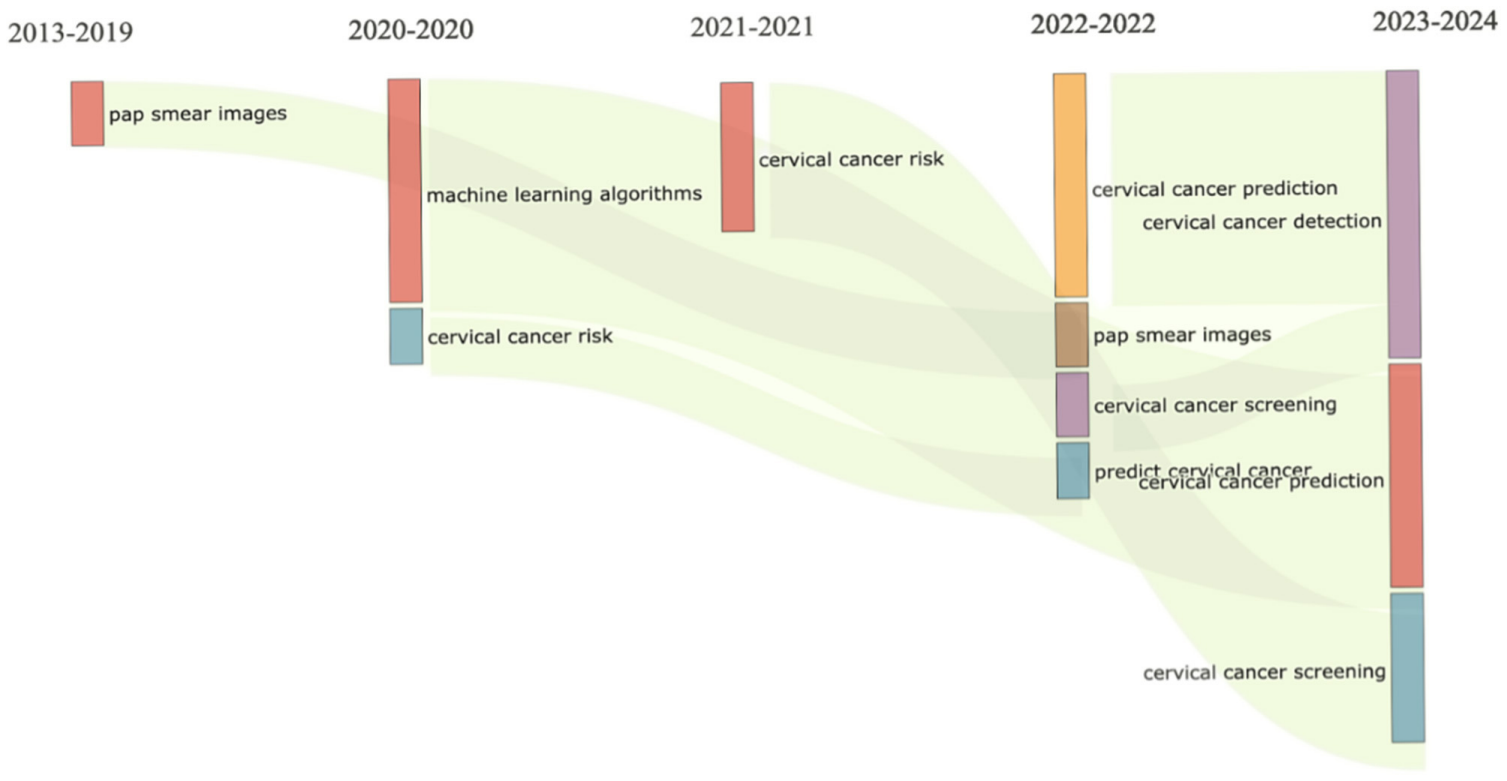
Thematic evolution.

The visualisation in Figure 5 revealed that between 2013 and 2019, research activities primarily focused on refining pap smear imaging techniques, a fundamental aspect of cervical cancer screening. This sustained emphasis on pap smear imaging highlights the pivotal role in identifying precancerous lesions and early-stage malignancies. Concurrently, investigations into cervical cancer risk spanned several years (2013–2021), reflecting ongoing efforts to elucidate risk factors, epidemiology, and preventive measures. Studies explored genetic predispositions, viral associations (such as human papillomavirus), and lifestyle factors contributing to cervical cancer risk, contributing to a comprehensive understanding of the disease's aetiology and prevention strategies.

The years 2020–2021 witnessed a significant shift with the emergence of machine learning algorithms in cervical cancer research. Researchers adopted artificial intelligence to enhance diagnostic accuracy and predictive models, analysing complex datasets comprising imaging results and patient histories for personalised risk assessment. Subsequently (2021–2022), predictive modelling gained prominence, aiming to forecast individualised outcomes such as disease progression, treatment response, and recurrence. Integrating clinical data with machine learning algorithms facilitated more precise risk stratification, marking a notable advancement in cervical cancer risk prediction and management strategies.

**Figure 6:**
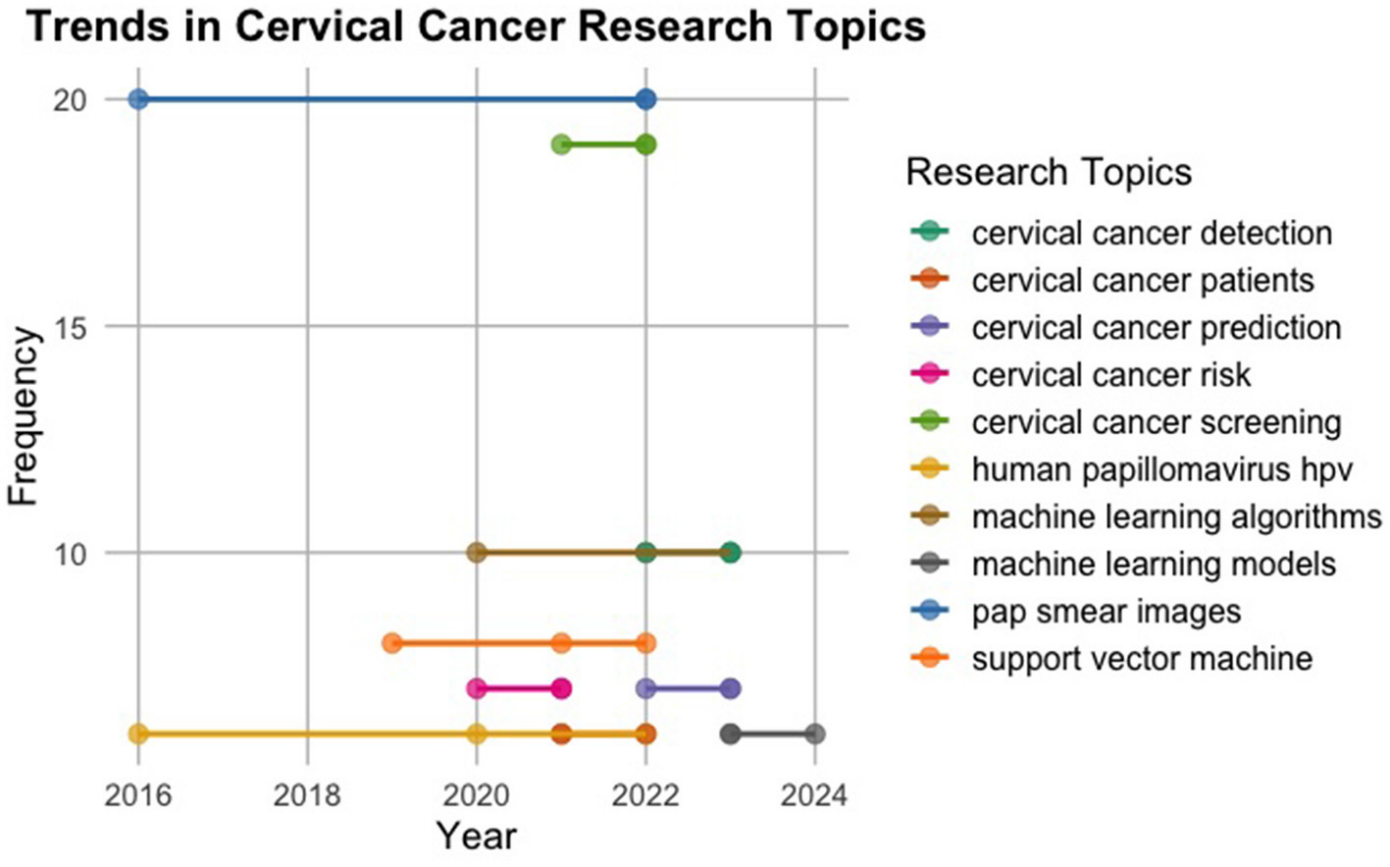
Trend topics.

The provided Figure 6 offers insight into the evolving landscape of topics related to cervical cancer and machine learning from 2016 to 2021. Each topic is depicted by blue dots, with their sizes indicating the term frequency, denoted by a legend on the right. Notable topics include cervical cancer detection, machine learning models, Pap smear images, cervical cancer screening, machine learning algorithms, support vector machine, cervical cancer risk, cervical cancer patients, and human papillomavirus (HPV). The graph highlights the progressive nature of research and development in cervical cancer detection, machine learning, and associated domains, emphasising the ongoing efforts to advance diagnostic and predictive capabilities for improved healthcare outcomes.

**Figure 7:**
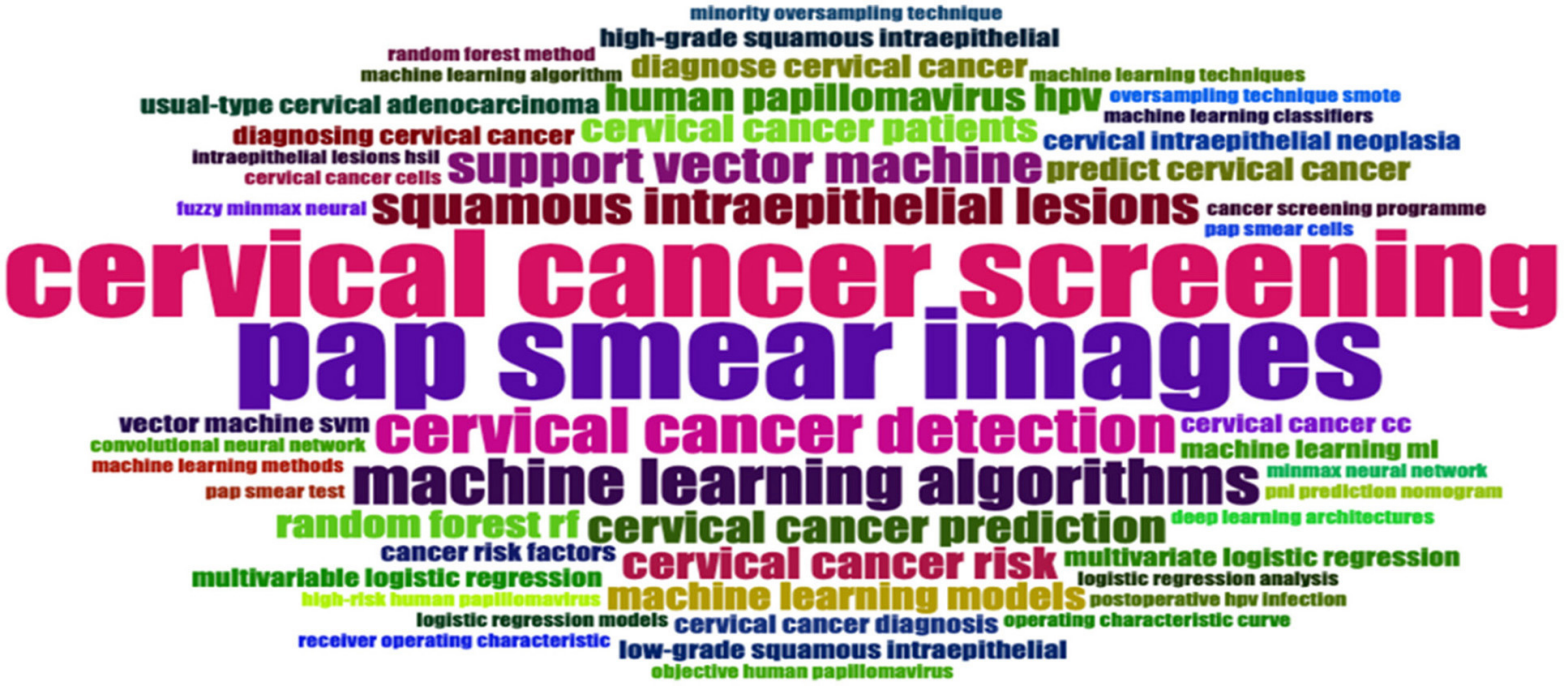
Word cloud.

The significance of the word cloud (see Figure 7) analysis lies in its ability to highlight key themes and trends within cervical cancer screening research. It reveals the central focus on cervical cancer screening, indicating the ongoing efforts to improve detection and diagnosis methods. The prominence of terms like “Pap Smear Images,” “Human Papillomavirus (HPV),” “Low-Grade Squamous Intraepithelial,” “High-Grade Squamous Intraepithelial,” and “Machine Learning Algorithms” suggests a shift towards incorporating advanced technology for more accurate and efficient screening processes.

This implies a potential transformation in clinical practise, with the adoption of innovative approaches to enhance early detection rates and improve patient outcomes. Terms like “Cervical Cancer Detection,” “Random Forest (RF),” and “Support Vector Machine” show the importance of early diagnosis and the specific techniques employed to achieve this goal. Overall, the word cloud analysis shows the evolving field of cervical cancer screening research, highlighting the integration of technology and the ongoing pursuit of improved screening methods for better patient care.

### 3.4 Bibliographic coupling

**Figure 8:**
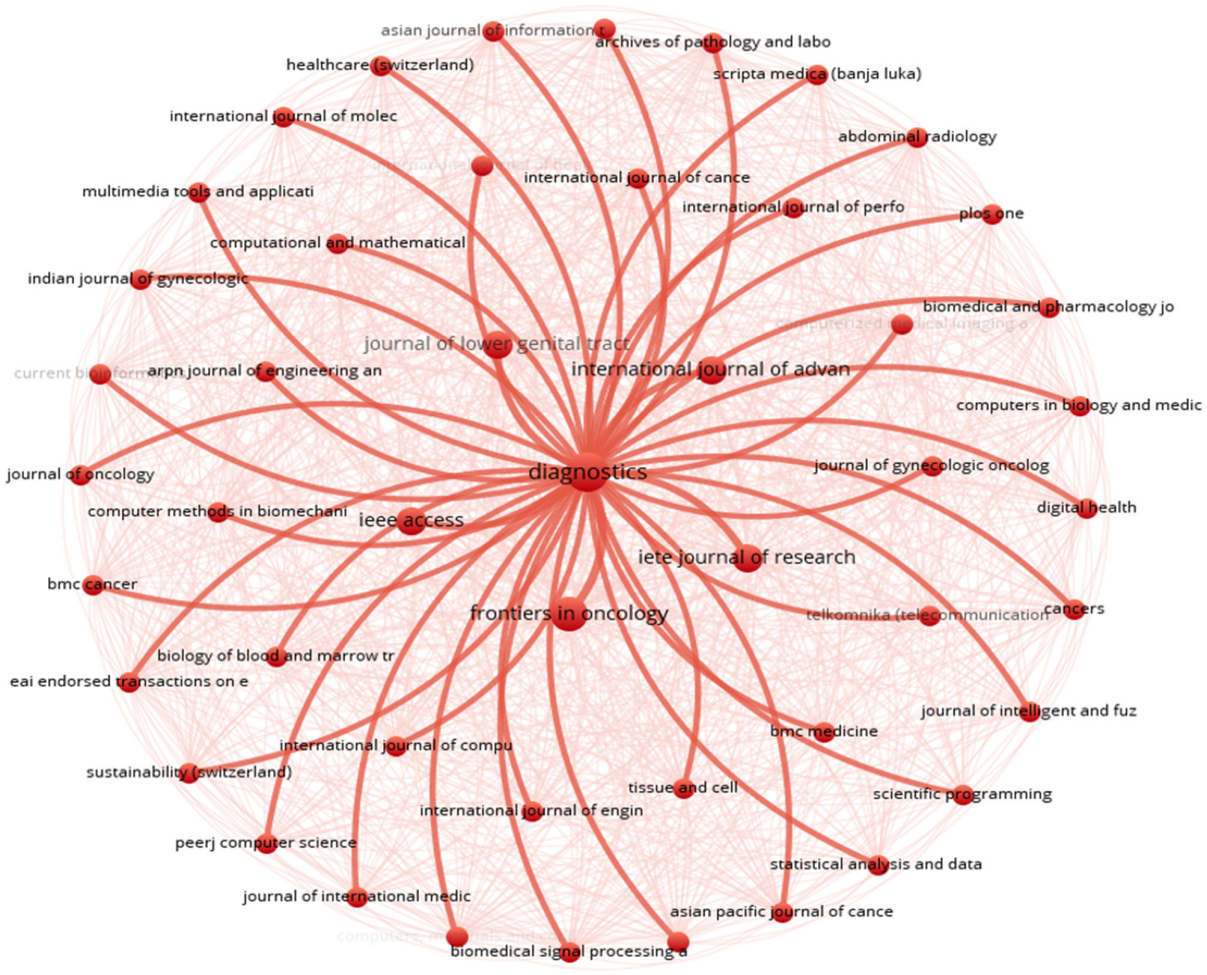
Bibliographic coupling.

A key finding was the recurring theme of “diagnostics” (see Figure 8). This theme appeared as a central element in the visualisation, highlighting its widespread relevance across the analysed articles. The prominence of diagnostics suggested its importance as a major area of research focus.

The visualisation also revealed a diverse range of journals and research fields connected to diagnostics. Each surrounding node represented a specific journal or field of study, such as the Asian Journal of Information Technology, Healthcare Technology Letters, International Journal of Molecular Sciences, Abdominal Radiology, Multimedia Tools and Applications, Computational Mathematics International Journal, and Indian Journal of Science & Technology. This variety of nodes showcased the broad scope of research areas associated with diagnostics.

Red lines connect the central “diagnostics” theme to other nodes, indicating bibliographic coupling. This means there were close relationships between diagnostics research and various research domains. These connections highlighted the interdisciplinary nature of diagnostics research, suggesting its interconnectedness with multiple fields. The visualisation provided evidence for the role diagnostics plays in integrating different areas of research.

This approach went beyond traditional citation analysis by uncovering interconnected relationships between research topics and journals, offering valuable insights into the multidisciplinary nature of diagnostics research. Unlike traditional citation analysis, which primarily focuses on citation counts and direct references, Figure 8 provided a more comprehensive view of the complex interplay between different areas of scholarly inquiry.

The findings demonstrated the collaborative nature of interdisciplinary research efforts in driving advancements in cervical cancer diagnosis by highlighting the interconnectedness between diagnostics research and various research domains. This deeper understanding emphasises the importance of interdisciplinary collaboration in addressing complex healthcare challenges and highlights the pivotal role of diagnostics research in advancing diagnostic capabilities for cervical cancer.

### 3.5 Scientific production

**Figure 9:**
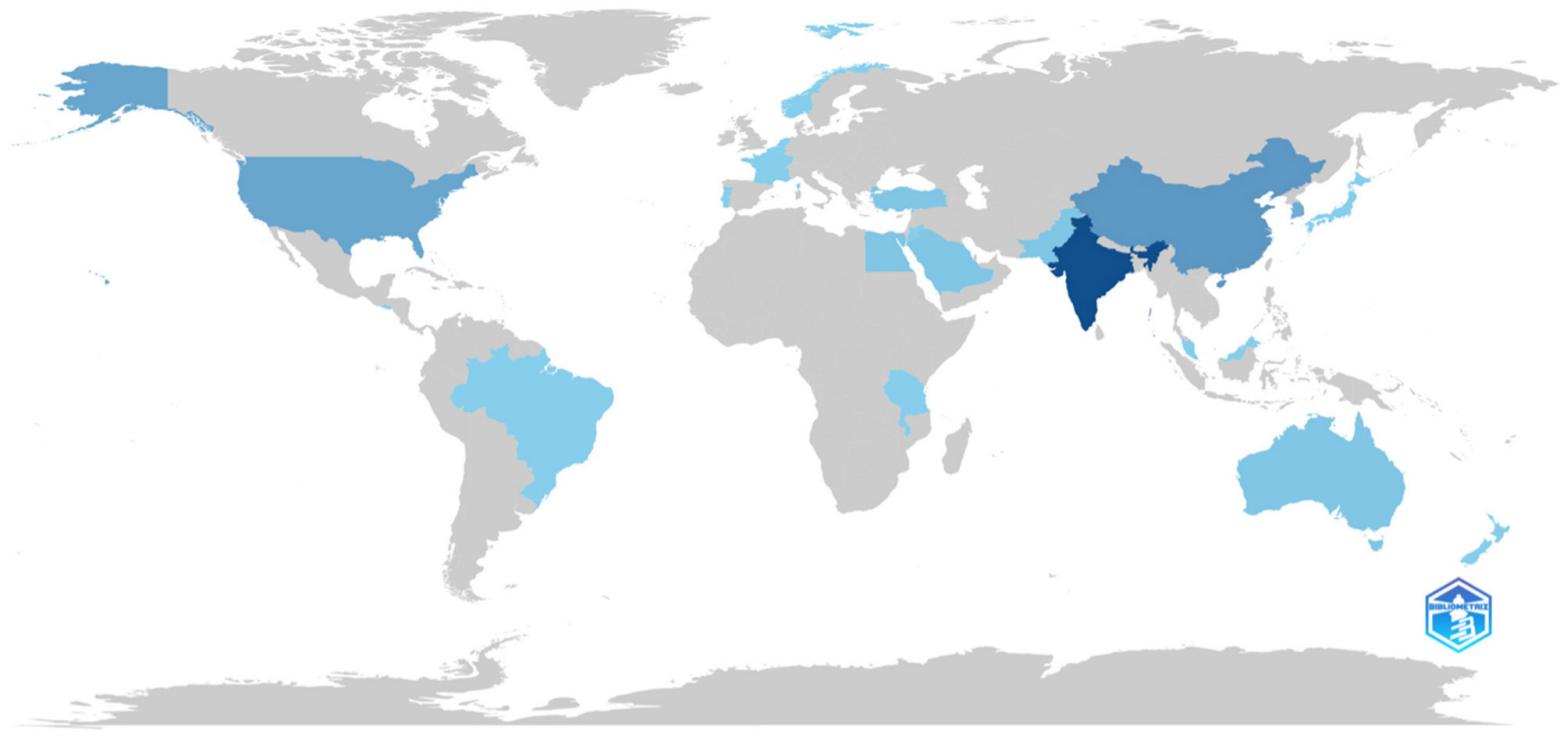
Map on country scientific production.

The map in Figure 9 illustrates the distribution of scientific output across different countries regarding predictive modelling for cervical cancer risk. Countries are shaded in varying shades of blue to denote the volume of publications, with darker shades indicating higher production. Notably, India, United States, China, and Australia emerge as significant contributors to this field. Conversely, Africa appears mostly unshaded, indicating minimal research output, underscoring the disparity in scientific contributions between developed nations and African countries.

This observation emphasises the necessity for heightened investment and focus on scientific research concerning cervical cancer risk prediction in African nations. Closing this research gap not only aids in addressing the burden of cervical cancer within Africa but also holds promise for enhancing screening, prevention, and treatment strategies on a global scale. Redirecting resources and support towards scientific activities in Africa can pave the way for achieving greater equity in healthcare access and mitigating the global burden of cervical cancer.

**Figure 10:**
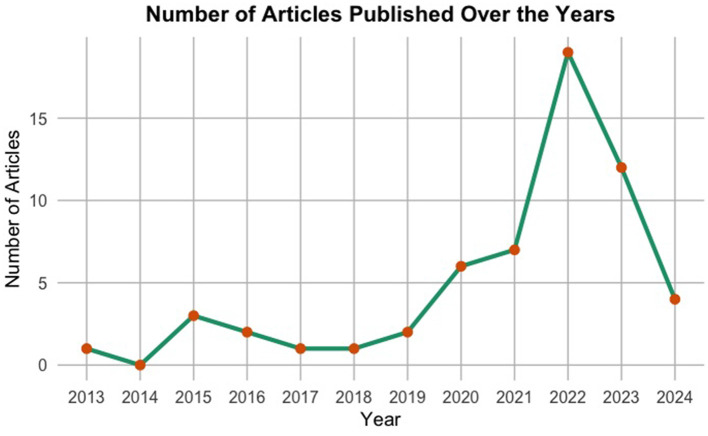
Annual scientific production.

The provided Figure 10 illustrates a succinct overview of the number of articles produced each year in the field of predictive modelling for cervical cancer risk. Spanning from ~2013 to 2023, the *x*-axis denotes the years, while the *y*-axis indicates the number of articles published. Notably, until around 2018, there was a steady production of fewer than five articles per year. However, post-2018, there is a notable surge in article production, reaching a peak in 2022 with over 15 articles. Subsequently, there is a sharp decline in 2022. This spike in 2021 indicates a surge in research activity, while the decline in 2022 may suggest a shift in research focus or external factors influencing publication trends.

This analysis of publication trends highlights the growing importance of predictive modelling in cervical cancer risk assessment, signifying its potential to improve preventative healthcare strategies. While the decline in 2023 publications requires further exploration, it highlights the dynamic nature of this research field. Continued monitoring of publication trends alongside a deeper understanding of the underlying reasons for these shifts can provide valuable insights for researchers and stakeholders invested in advancing this critical field.

## 4 Limitations

Limitations of this study include the exclusive reliance on the Scopus and Web of Science databases, potentially omitting relevant studies from other sources. Our search encompassed all literature on predictive modelling for cervical cancer risk. However, scholarly attention to this topic became prominent only from 2013 onwards. No literature predating 2013 addressed predictive modelling for cervical cancer risk, leading to the retrieval of relevant articles exclusively from 2013 onwards.

## 5 Conclusion

This study aimed to identify literature and thematic content in cervical cancer risk prediction modelling through citation analysis and to explore research trends, collaboration patterns, and niche areas. The study significantly enhanced the understanding of cervical cancer risk prediction by integrating Braun and Clarke's framework with NLP and LDA. It provided insights into core themes, relationships, and broader trends, offering a solid foundation for future research and improvements in cervical cancer prevention, diagnosis, and treatment.

Integrating diverse expertise from fields such as mathematical disciplines, biomedical health, healthcare practitioners, public health and policy is essential for a comprehensive approach to cervical cancer risk prediction. This interdisciplinary collaboration leads to more robust and holistic solutions. The adoption of advanced machine learning algorithms, transitioning from simpler models like logistic regression to more complex algorithms such as random forest and support vector machines, significantly enhances the accuracy and efficiency of cervical cancer detection and predictive modelling.

These advancements are crucial for early detection and improved patient outcomes, which are vital for effective public health interventions. However, many studies lack external validation on independent datasets, limiting the robustness and generalisability of their findings. Furthermore, addressing model interpretability is crucial for understanding prediction mechanisms and ensuring that healthcare providers can trust and effectively use these models in clinical settings.
